# Joint pain severity predicts premature discontinuation of aromatase inhibitors in breast cancer survivors

**DOI:** 10.1186/1471-2407-13-401

**Published:** 2013-09-03

**Authors:** Kannie Chim, Sharon X Xie, Carrie T Stricker, Qing S Li, Robert Gross, John T Farrar, Angela DeMichele, Jun J Mao

**Affiliations:** 1 Department of Family Medicine and Community Health, University of Pennsylvania, 3400 Spruce Street - 2 Gates, Philadelphia, Pennsylvania 19104, USA; 2 Center for Clinical Epidemiology and Biostatistics, Perelman School of Medicine at the University of Pennsylvania, Philadelphia, PA 19104, USA; 3 Abramson Cancer Center, Perelman School of Medicine at the University of Pennsylvania, Philadelphia, PA 19104, USA; 4 Department of Medicine, Perelman School of Medicine at the University of Pennsylvania, Philadelphia, PA 19104, USA

**Keywords:** Aromatase inhibitor, Joint pain, Adherence, Adverse effects, Musculoskeletal, Breast cancer, Pain diagnosis, Pain management, Survivorship

## Abstract

**Background:**

Premature discontinuation of aromatase inhibitors (AIs) in breast cancer survivors compromises treatment outcomes. We aimed to evaluate whether patient-reported joint pain predicts premature discontinuation of AIs.

**Methods:**

We conducted a retrospective cohort study of postmenopausal women with breast cancer on AIs who had completed a survey about their symptom experience on AIs with specific measurements of joint pain. The primary outcome was premature discontinuation of AIs, defined as stopping the medication prior to the end of prescribed therapy. Multivariate Cox regression modeling was used to identify predictors of premature discontinuation.

**Results:**

Among 437 patients who met eligibility criteria, 47 (11%) prematurely discontinued AIs an average of 29 months after initiation of therapy. In multivariate analyses, patient-reported worst joint pain score of 4 or greater on the Brief Pain Inventory (BPI) (Hazard Ratio [HR] 2.09, 95% Confidence Interval [CI] 1.14-3.80, *P* = 0.016) and prior use of tamoxifen (HR 2.01, 95% CI 1.09-3.70, *P* = 0.026) were significant predictors of premature discontinuation of AIs. The most common reason for premature discontinuation was joint pain (57%) followed by other therapy-related side effects (30%). While providers documented joint pain in charts for 82% of patients with clinically important pain, no quantitative pain assessments were noted, and only 43% provided any plan for pain evaluation or management.

**Conclusion:**

Worst joint pain of 4 or greater on the BPI predicts premature discontinuation of AI therapy. Clinicians should monitor pain severity with quantitative assessments and provide timely management to promote optimal adherence to AIs.

## Background

Third-generation aromatase inhibitors (AIs) are commonly prescribed as standard adjuvant therapy for postmenopausal breast cancer survivors with hormone-receptor positive disease and are associated with improved disease-free survival compared with the previous standard of tamoxifen therapy [1-4]. The recommended duration of initial adjuvant endocrine therapy is five years though some patients have benefitted from extended therapy. Current American Society of Clinical Oncology guidelines recommend incorporating an AI either as primary, sequential (following 2–3 years of tamoxifen), or extended therapy (following 5 years of tamoxifen) [5].

Despite the survival benefits of AIs, many women demonstrate some degree of non-adherence with their use. Non-adherence, a term which comprises both non-compliance with dosing, timing, and instruction of medication and non-persistence, or early discontinuation of medication, represents an emergent area to intervene for treatment benefit. Indeed, non-adherence of adjuvant endocrine therapy has been associated with increased mortality in breast cancer patients [6]. In the emerging literature, premature discontinuation of AI therapy ranges from 13-35% [7-9].

Despite the prevalence of early medication discontinuation and its deleterious effects, little is understood about risk factors for premature discontinuation of AI therapy. The available adherence research largely consists of medical claims-based epidemiological studies in which younger age, increased cormorbidities, and higher medication co-payments have been noted as risk factors for nonadherence to adjuvant therapy [10-12]. While these studies are useful in quantifying the magnitude of medication nonadherence, they often lack clinical insights into patient perspectives such as the reasons for premature discontinuation. AI-associated arthralgia, or joint pain, has been recognized as a particularly debilitating side effect which develops in nearly half of women treated with AIs [13,14]. In online breast cancer message board discussions, joint pain is the most commonly mentioned side effect of AIs and often leads to AI discontinuation [15]. Although joint pain was the most cited reason for premature discontinuation of AIs in a recently published clinical trial, little is known about how levels of joint pain may predict early discontinuation of therapy [9]. Appropriate identification of patients most at risk for discontinuing therapy may provide an opportunity for early interventions to alleviate the adverse effects of joint pain and improve medication adherence. We conducted a retrospective cohort study to determine whether patient-reported joint pain severity predicts premature discontinuation of AIs. As a secondary aim, we examined provider-documented pain and practice behaviors among those with clinically important pain.

## Methods

### Study design and patient population

Participants were identified from the Wellness after Breast Cancer (WABC) study, an ongoing cohort study of breast cancer patients who completed a survey at the time of recruitment between March 2008 and July 2009 at the University of Pennsylvania (Philadelphia, PA, USA) [16]. The inclusion criteria for the WABC study were: (1) postmenopausal status (amenorrhea ≥ 12 months), (2) histologically-confirmed stage I-III hormone receptor-positive breast cancer, (3) exposure to a third-generation AI (anastrozole, letrozole, or exemestane), (4) completion of all chemotherapy and/or radiotherapy at least one month prior to survey date, (5) approval of the patient’s primary oncologist, and (6) ability to provide informed consent. Participants had been on AI therapy on average 26.7 months at time of entry to the WABC cohort. Participants were approached while in the waiting room for their oncology appointments by trained research assistants. After informed consent was obtained, each participant completed a self-administered survey. We performed a retrospective cohort study from October - December 2011 of all women enrolled in the WABC study who were taking an AI at the time of survey as determined by chart review. Detailed chart abstraction was performed to measure study related outcomes and variables. The study was approved by the Institutional Review Board of the University of Pennsylvania.

### Primary outcome: premature discontinuation

Premature discontinuation of AIs, defined as stopping medication prior to the end of prescribed therapy, was determined by chart review of outpatient electronic medical records (EMR). Medication events were evaluated from the survey date to December 2011 (follow-up period of 29–45 months). Each oncology visit progress note was reviewed for information concerning the prescribed AI and planned duration of therapy at the time of prescription. Patients varied in their duration of therapy depending on whether an AI was incorporated as primary, sequential, or extended adjuvant endocrine therapy and we deferred to the provider-determined end date for all patients as documented in the EMR [5]. AI switches and drug holidays were not regarded as premature discontinuation events unless the patient ultimately discontinued their second AI or did not resume AI therapy after a drug holiday. For those who stopped therapy early, the reasons for premature discontinuation were abstracted from the EMR on the date of the clinical visit.

We regarded premature discontinuation as an intentional action of the patient in line with the definition of non-persistence presented by Guth *et al*. [17]. Discontinuations of therapy due to breast cancer recurrence were not considered premature discontinuation events. We censored subjects at the time of disease recurrence (*N*=5), death (*N*=8), or loss to follow up from the outpatient clinic (*N*=20).

### Patient-reported joint pain

Patient-reported pain outcomes were obtained from the one time baseline survey that established the cohort. To evaluate clinically important joint pain, we used the worst pain measure from the Brief Pain Inventory (BPI) with slight modifications [18]. Participants were asked to rate their worst joint pain in the past 24 hours on a scale of 0 (no pain) to 10 (pain as bad as you can imagine). We *a priori* dichotomized patients into two groups: those reporting joint pain severity from 0–3 and those reporting joint pain from 4–10, a level at which pain becomes clinically important and interferes with daily functioning [19]. To evaluate the presence of AI-related arthralgia (AIAA), women were first asked if they were experiencing joint pain. They were then asked to specify the perceived source of their arthralgia: “prior osteoarthritis; aromatase inhibitors; aging; weight gain; other medical conditions; other medications; others; I don’t have joint symptoms.” Respondents were able to choose more than 1 option. Consistent with our prior research, patients who selected “aromatase inhibitors” were considered to have AIAA [14].

### Covariates

Self-reported demographic variables included age, race/ethnicity, education level, date of last menstrual period (LMP), and reasons for menopause (natural or induced). Comorbidities were assessed using a standard checklist and categorized into 0, 1, or 2, or more conditions. Clinical variables such as tumor type, stage, treatment regimen, and treatment status were collected via medical chart abstraction.

### Secondary outcome: clinician documentation of joint pain

Provider encounter notes in the EMR on the date each subject completed the initial WABC survey were reviewed to compare provider and patient reports of joint pain. We analyzed the visit note for documentation of joint pain and, if present, indications of the level of joint pain using quantitative pain ratings and whether a plan to address joint pain was provided.

### Statistical analysis

Data analysis was conducted using STATA 12 for Windows (STATA Corporation, College Station, TX). Survival analyses were performed using the Kaplan-Meier method to examine individual predictors of premature discontinuation from the time of initial survey. Multivariate Cox proportional hazards regression models were used to estimate the association between predictive variables (those variables that were associated with the outcome in bivariate analyses with *P* <0.10) and premature AI discontinuation. All statistics were two-sided with *P* <0.05 indicating significance.

## Results

### Patient characteristics

**Figure 1:**
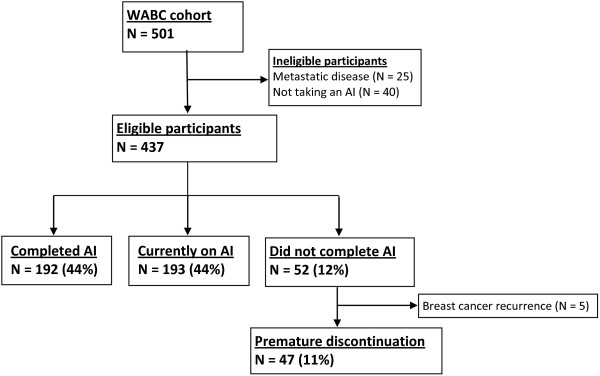
Patient selection and follow up.

Of 501 subjects enrolled in the WABC study, 461 (92%) were taking an AI at survey date. Twenty-four subjects (4.8%) were excluded after chart review revealed metastatic disease at the time of enrollment, leaving a total of 437 eligible patients (Figure 1). Among these subjects (Table 1), the mean age was 62 years (standard deviation 10.2). Although the majority of patients (82%) was non-Hispanic white, a substantial proportion (15%) was non-Hispanic black. In the analysis, we combined the race categories into white and nonwhite. More than three-quarters of participants had a college education or greater (343 subjects, 79%) with 21% reporting high school or less. Regarding prior treatment, 268 (61%) had undergone chemotherapy (see Table 1 for taxane vs. non-taxane regimens) and 147 (34%) reported prior use of tamoxifen. The majority of patients (81%) reported taking anastrozole. A third of subjects (156; 36%) met criteria for clinically important pain with worst joint pain rating between 4–10 in the past 24 hours and nearly half of all subjects (206; 47%) reported joint symptoms attributable to AIs (Table 1).

**Table 1:** Characteristics of study participants

	**N**	**%**	**HR (95% CI)**	***P*-value**
**Total (N, %)**	437	100.0		
**Age, years**				0.26
>65	138	31.6	1	
55-65	201	46.0	0.58 (0.30-1.13)	
<55	98	22.4	0.81 (0.39-1.68)	
**Race**				0.51
White	360	82.4	1	
Non-white	77	17.6	0.77 (0.34-1.72)	
**Education**				0.071
College or above	343	78.7	1	
High school or less	93	21.3	0.42 (0.17-1.08)	
**Years since LMP**				0.59
>10	242	56.1	1	
5-10	108	25.1	1.42 (0.72-2.77)	
<5	81	18.8	1.05 (0.48-2.29)	
**Reasons for menopause**				
Natural	226	52.4	1	
Induced	205	47.6	1.44 (0.81-2.56)	
**Comorbid conditions**				0.35
None	68	15.6	1	
One	133	30.4	0.84 (0.38-1.89)	
Two or more	236	54.0	0.59 (0.27-1.30)	
**Stage**				0.46
I	169	38.6	1	
II	214	49.0	0.77 (0.42-1.41)	
III	54	12.4	0.56 (0.19-1.63)	
**Chemotherapy**				0.92
None	169	38.7	1	
Chemotherapy but no taxane	104	23.8	0.92 (0.41-2.05)	
Chemotherapy included taxane	164	37.5	1.08 (0.57-2.04)	
**Years since start of AI**				0.66
<1	139	31.8	1	
1-3	145	33.2	0.74 (0.39-1.43)	
>3	153	35.0	0.81 (0.38-1.76)	
**Prior tamoxifen use**				0.088
No	290	66.4	1	
Yes	147	33.6	1.70 (0.94-3.08)	
**Aromatase inhibitor**				0.22
Anastrozole	299	70.1	1	
Letrozole	80	18.7	0.57 (0.25-1.30)	
Exemestane	47	11.0	0.98 (0.37-2.56)	
**Patient-reported worst joint pain**				**0.037**
0-3	277	64.0	1	
4-10	156	36.0	1.86 (1.04-3.31)	
**Patient-reported joint pain related to AIs**				0.14
No	231	52.9	1	
Yes	206	47.1	1.55 (0.87-2.76)	

*Abbreviations*: *HR* hazard ratio, *95% CI* 95% confidence interval, *LMP* last menstrual period, *AI* aromatase inhibitor.

### Premature discontinuation

Among the cohort, 192 (44%) had completed their course of AI therapy for the full duration prescribed, while 193 (44%) continued to take an AI. Forty-seven women (11%) prematurely discontinued their course of AI therapy after an average of 29.4 ± 18.2 months (Figure 1). The most common reason for premature discontinuation recorded by providers in the EMR was joint pain (57%) followed by other therapy-related side effects (30%) and non-therapy-related side effects/unknown (13%). Five patients ceased AI therapy due to breast cancer recurrence.

### Predictors of premature discontinuation

**Figure 2:**
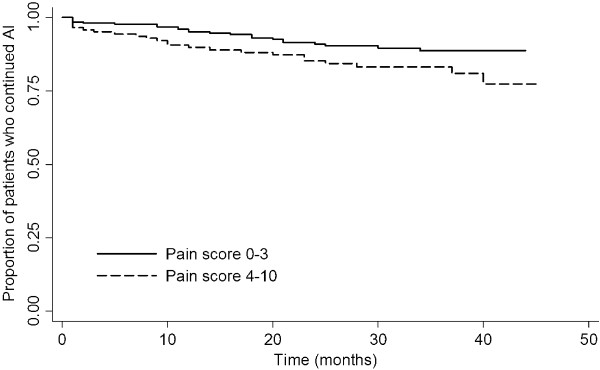
Kaplan-Meier curve comparing continuation of AI therapy between patients reporting worst joint pain score on Brief Pain Inventory of 0-3 with those reporting worst pain of 4-10 (clinically important pain).

In univariate analyses, patient-reported joint pain severity ≥4 (measured at cohort entry) was significantly associated with premature discontinuation of AI therapy (*P* = 0.037). Higher education level (*P* = 0.071) and prior use of tamoxifen (*P* = 0.088) were also associated with early discontinuation but did not reach statistical significance (Table 1). In the multivariate regression model, adjusting for variables selected from the univariate analyses, joint pain severity and prior use of tamoxifen were statistically significant independent predictors of premature discontinuation (Table 2). Women reporting joint pain severity of 4 or greater were more likely to stop AI therapy early than women reporting joint pain of 3 or less (Hazard Ratio [HR] 2.09, 95% Confidence Interval [CI] 1.14-3.80, *P* = 0.016) (Figure 2). Prior tamoxifen use was also significantly associated with premature discontinuation of AI therapy (HR 2.01, 95% CI 1.09-3.70, *P* = 0.026).

**Table 2:** Predictors of premature discontinuation of aromatase inhibitors

	**Univariate analyses**			**Multivariate analysis**		
**Predictors**	**HR**	**95% CI**	***P*-value**	**HR**	**95% CI**	***P*-value**
**Patient-reported worst joint pain**						
0-3	1			1		
4-10	1.86	1.04-3.31	**0.037**	2.09	1.14-3.80	**0.016**
**Prior tamoxifen use**						
No	1			1		
Yes	1.7	0.94-3.07	0.088	2.01	1.09-3.70	**0.026**
**Education**						
College and above	1			1		
High school or less	0.42	0.17-1.08	0.071	0.37	0.13-1.03	0.057

*Abbreviations*: *HR* hazard ratio, *95% CI* 95% confidence interval.

### Clinician documentation of joint pain

We restricted our analysis of provider behavior to include only individuals reporting clinically important pain (worst joint pain rating 4–10) on the initial survey (*N* = 151). Provider (e.g., MD or NP) documentation of joint pain was noted in 124 visits (82%), however, no quantitative assessments of pain were observed. Providers documented a plan for joint pain (e.g., imaging, specialist referral, return if pain worsens) in 43% of visits.

## Discussion

In this retrospective analysis we evaluated whether patient-reported joint pain severity is predictive of premature discontinuation of AIs. After controlling for demographic and clinical characteristics, patients who reported worst joint pain severity of 4 or greater on the BPI were significantly more likely to prematurely discontinue AI therapy. However, patient-reported AIAA did not predict premature discontinuation. Although clinicians often documented the presence of joint pain in the EMR of patients with clinically important pain, no quantitative assessment was used, and only half offered a plan to further evaluate or treat pain. These findings warrant future clinical improvements in both pain diagnosis and management for breast cancer survivors receiving AI therapy.

Premature discontinuation specific to AI therapy has been reported to range from 13-35% in the emerging literature [7-9]. These variations may be due to population characteristics (clinical trial vs. community), time to follow-up, and different definitions of premature discontinuation. In our study 11% of women who were on AI therapy at cohort entry stopped medication during a follow-up period of 29–45 months. This rate is predictably lower than the total premature discontinuation rate of the entire cohort given that nearly half of our study population was still taking an AI at the time of the study and remain at risk for prematurely discontinuing therapy. Additionally, 40 individuals were excluded from analysis for not taking AIs at the time of recruitment. Among those, 29 patients had prematurely discontinued AI therapy; in sum, 16% of the sample which falls in the range of reported premature discontinuation rates in the literature.

Premature discontinuation, as one aspect of non-adherence, has substantial negative effects by the very meaning of its definition – the complete cessation of therapy. Hershman *et al*. demonstrated an absolute increase in mortality of more than 7% in women who prematurely discontinued hormonal therapy with either an AI or tamoxifen compared to those who continued therapy over a 4.5 year period. Even with adjustment for relevant clinical and demographic variables, early discontinuation was associated with a relative 26% increase in all-cause mortality [6]. These results emphasize the importance of identifying patients most at risk for stopping AIs and targeting interventions toward this group.

We found patient-reported worst joint pain severity of 4 or greater to be predictive of premature AI discontinuation. Interestingly, patient-reported AI-associated arthralgia was not a significant predictor, suggesting that it is the severity of joint pain, rather than its assumed origins, that may lead women to stop therapy. The finding of joint pain severity as a predictor of premature discontinuation is consistent with a recently published study in a clinical trial setting in which higher baseline pain is a positive predictor of early discontinuation of AI therapy [9]. These findings suggest that although patients may function reasonably well at low pain levels, a threshold may exist beyond which pain is difficult to ignore and may impact adherence behavior. Our results suggest a BPI worst pain rating of 4 or greater may be a threshold for predicting premature discontinuation to AI therapy, a finding warranting validation in independent datasets.

In contrast to research suggesting physicians' failure to routinely assess patient pain [20], it is encouraging that joint pain was documented in the majority of provider notes for patients reporting pain ratings of 4 or greater. Despite the high percentage of symptom reporting, the lack of specific pain quantification warrants improvement. Quantitative pain assessments provide a reliable and valid measure of pain intensity and are recommended by the National Comprehensive Cancer Network (NCCN) Practice Guidelines for cancer pain [21]. A simple measure such as a 10-point numerical rating scale of pain can be easily and reliably incorporated into an outpatient visit [20]. With the emergence of health technology, electronic patient-report assessments can be utilized to provide quantitative assessments of patient pain and facilitate patient-provider communication regarding symptom distress [22]. Ultimately, with targeted therapies currently under investigation to alleviate joint pain related to AIs [23-25], patients and their providers will have increasingly effective options to manage joint pain rather than discontinuing AI therapy.

In multivariate modeling, we found that controlling for joint pain severity strengthened the association between prior tamoxifen use and premature discontinuation of AIs. This suggests that for patients who experience the same level of pain severity, they are more likely to discontinue AIs if they had prior use of tamoxifen. Based on clinical experience, it is possible that prior tamoxifen users who perceived that they had adequate hormonal therapy were therefore less tolerant of side effects of AIs. Another possible explanation is that prior tamoxifen users have gotten used to the side effects of tamoxifen over time and when they experience new side effects related to AIs, they prefer to switch back to tamoxifen rather than staying on AIs.

Our study has several potential limitations. First, in our retrospective cohort study design, we assessed joint pain one time to predict premature discontinuation of AIs. As pain may change over time, our data does not provide an absolute cut-off pain level necessary to discontinue AIs and requires validation in independent datasets. Additionally, our retrospective design did not include patients who prematurely discontinued AIs prior to enrollment which may bias our results towards null. Thus, the strength of association between joint pain severity and premature discontinuation may in fact be even stronger. Third, our outcome focused on premature discontinuation based on chart-abstraction. Theoretically, patients may discontinue therapy without informing their providers; however, if that is the case, it should bias our results towards null. Lastly, our research was conducted in a large academic medical center which may limit the ability to generalize findings to community hospital settings.

## Conclusions

Despite these limitations, our study had a number of strengths including a large clinical population, incorporation of patient-reported outcome, and examination of provider pain diagnosis and management behaviors. We found that patient-reported joint pain severity of 4 or greater was significantly associated with early discontinuation of therapy. These results highlight the importance of medical providers performing quantitative assessments of pain and inquiring about other medication adverse effects in order to appropriately identify patients who may be at risk for stopping therapy. Targeting interventions for these patients will ultimately optimize adherence to AIs and improve both quality of life and survival outcomes for women with breast cancer.

## Consent

Written informed consent was obtained from each participant.

## Competing interests

RG has served on a Pfizer Data and Safety Monitoring Board for a drug unrelated to the treatment of breast cancer. JTF has consulted with Pfizer and AstraZeneca on design of clinical trials of pain therapeutics that are unrelated to aromatase inhibitors. AD has consulted and received research grants from Pfizer in the area of breast cancer. JM has consulted with Pfizer on pain medication unrelated to aromatase inhibitors or breast cancer.

## Authors’ contributions

KC designed the study, collected data come and drafted the manuscript. SX designed and performed statistical analyses. QSL performed statistical analyses and drafted the manuscript. CS, RG, JF, and AD participated in study design, assisted in data interpretation, and helped to draft the manuscript. JJM designed the study, drafted the manuscript, and obtained funding. All authors read and approved the final manuscript.

## Pre-publication history

The pre-publication history for this paper can be accessed here:

http://www.biomedcentral.com/1471-2407/13/401/prepub
